# Supplementary material for: Methamphetamine Induces the Release of Proadhesive Extracellular Vesicles and Promotes Syncytia Formation: A Potential Role in HIV-1 Neuropathogenesis
Source: Viruses. 2022 Mar 7;14(3):550. doi: 10.3390/v14030550 (PMC8950763; doi:10.3390/v14030550)
Supplement: Supplementary file 1 [file viruses-14-00550-s001.zip › viruses-1582097-supplementary.pdf]

viruses

MDPI

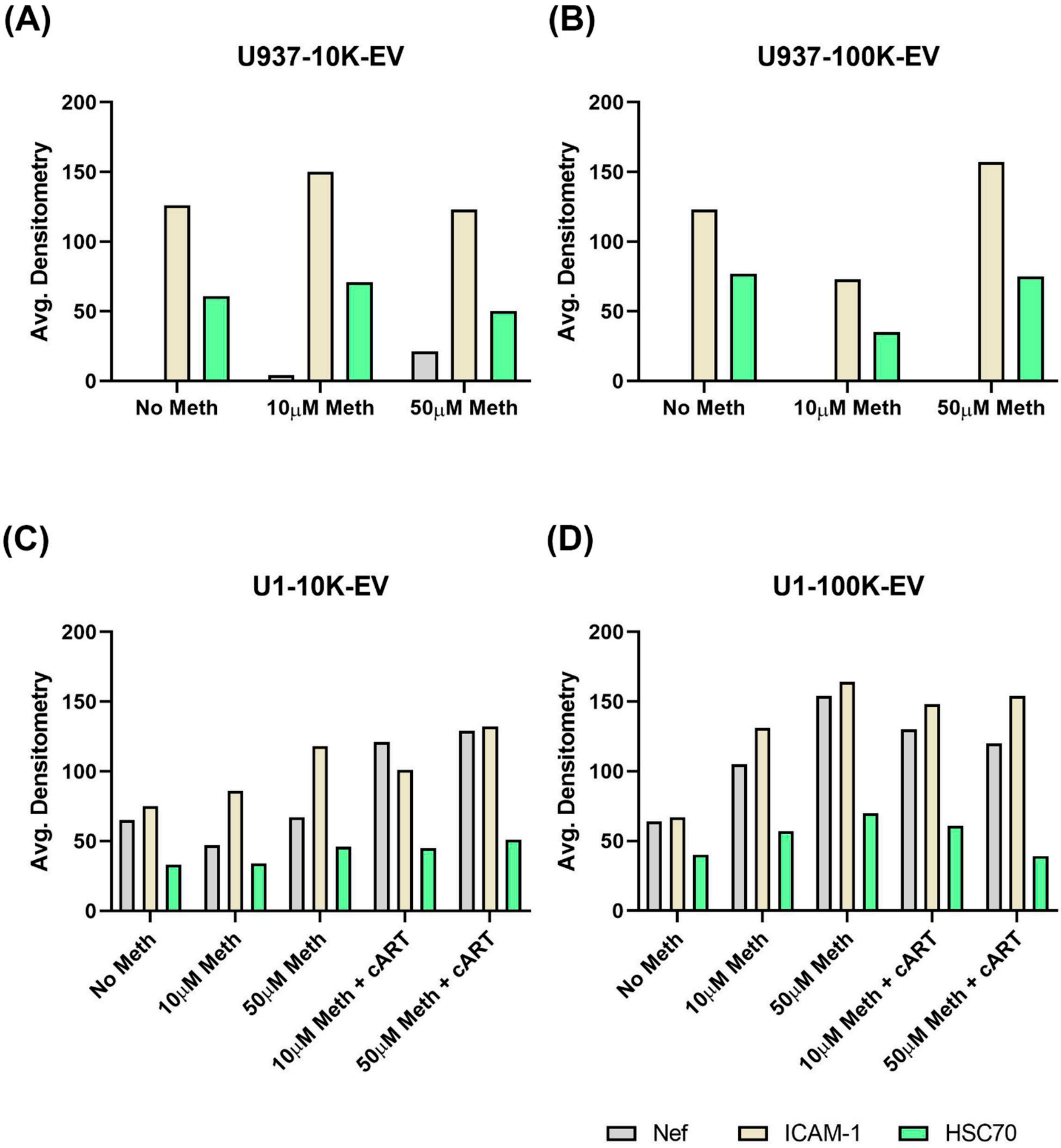

Supplementary Figure S1. Western blot quantification of different proteins in various groups of EVs. Average densitometry values from two independent experiments were taken into consideration for U937 10K (A), U937 100K EV (B), U1 10K EV (C), and U1 100K EV (D).

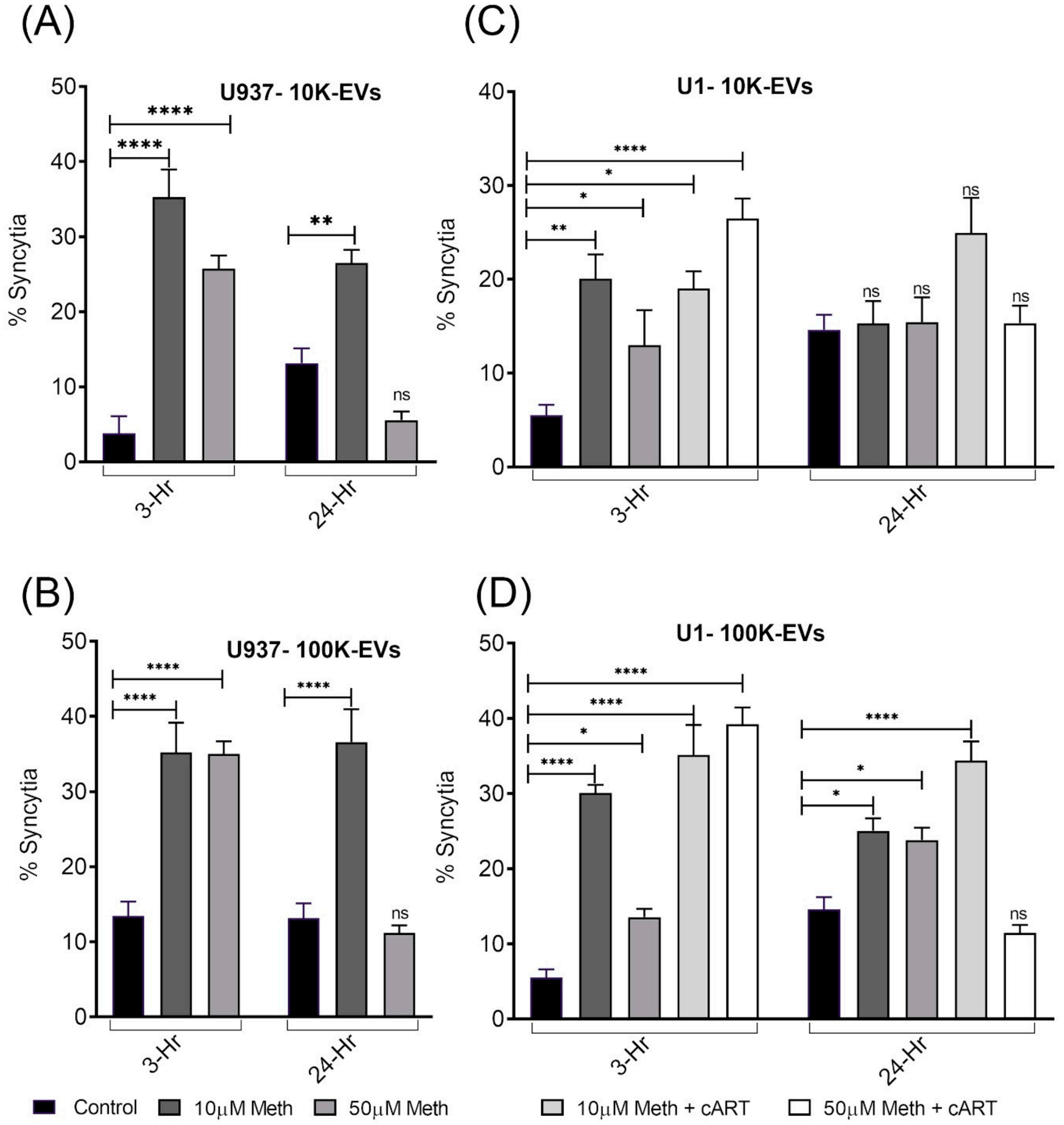

Supplementary Figure S2. Syncytia count induced by various groups of EV at 3 h and 24 hr. % of syncytia formation in the 10K EV set of different U937 groups for 3 and 24 h (A), 100K EV set of different U937 groups for 3 and 24 h (B), 10K EV set of different U1 groups for 3 and 24 h (C), and 100K EV set of different U1 groups for 3 and 24 h (D). Data are shown as mean +/- SEM and represent at least three independent assays done in triplicate. *p<0.05; **p<0.01; ***p<0.001, ****p<0.0001, and ns = not significant.
